# Supplementary material for: The cAMP Receptor Protein (CRP) of Vibrio mimicus Regulates Its Bacterial Growth, Type II Secretion System, Flagellum Formation, Adhesion Genes, and Virulence
Source: Animals (Basel). 2024 Jan 29;14(3):437. doi: 10.3390/ani14030437 (PMC10854923; doi:10.3390/ani14030437)
Supplement: Supplementary file 1 [file animals-14-00437-s001.zip › animals-2797153-supplementary.pdf]

Supplementary Material

The cAMP Receptor Protein (CRP) of *Vibrio mimicus* Regulates its Bacterial Growth, Type II secretion system, Flagellum Formation, Adhesion Genes and Virulence

Ziqi Tian [1,†], Fei Xiang [1,2,†], Kun Peng [1], Zhenyang Qin [1], Yang Feng [1], Bowen Huang [1], Ping Ouyang [1], Xiaoli Huang [3], Defang Chen [3], Weimin Lai [1], Yi Geng [1,*]

[1] College of Veterinary Medicine, Sichuan Agricultural University, Chengdu 611130, China

[2] Agricultural and Rural Bureau of Zhongjiang County, Deyang 618100, China

[3] Department of Aquaculture, Sichuan Agricultural University, Chengdu 611130, China

† These authors contributed to this work equally.

*** Correspondence:**

Corresponding Author: Yi Geng

Address: Huimin Road No. 211, Chengdu, Sichuan Province, Chengdu 611130, China

Tel.: +86028-86291162

E-mail: gengyisicau@126.com

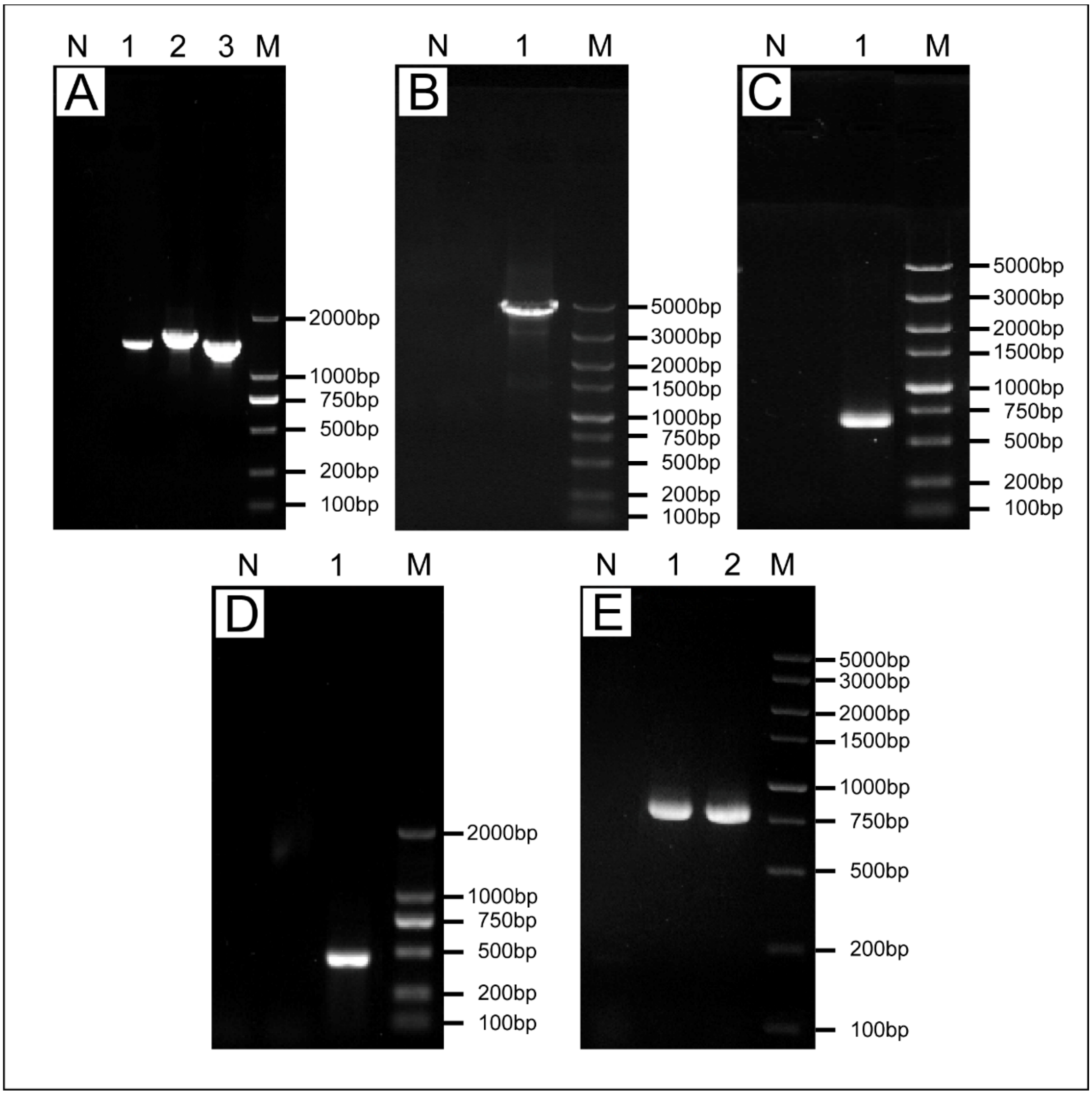

Fig S1. Construction and verification of the deletion and the complementation strains. A: Homologous recombination fragments amplified by primer pairs Up-F/R, Down-F/R and Kan^r-F/R, respectively (1: Upstream homology arm fragment, 2: *Kan*^{r} gene fragment, 3: Downstream homology arm fragment); B: Fusion fragment; C: Primer pair pKD46-F/R for identification of SCCF01/pKD46; D: Primer pair pCP20-F/R for identification of pCP20 plasmid; E: Primer pair pBAD24-F/R for identification of complementing strain; N: negative control; M: DNA Marker.

Table S1. Construction and detection Primers used in this study.

Primer	Primer sequences(forward/reverse,5′-3′)	Purpose
Up	F: ATCCTCAATGCACAATCCCAT<br>R: CGAAGCAGCTCCAGCCTACACTTGCTTGTATCACACGGCAAC	Amplification of the 1378 bp DNA fragment upstream of the 633 bp target deletion region
Down	F: ATTCATATGGACCATGGCTAAGCTATCCATATTTTAGCGAAGCC<br>R: ACTCGACAATTTGATCCGTGA	Amplification of the 1347 bp DNA fragment downstream of the 633 bp target deletion region
Kan^r	F: GTTGCCGTGTGATACAAGCAAGTGTAGGCTGGAGCTGCTTCG<br>R: GGCTTCGCTAAAATATGGATAGCTTAGCCATGGTCCATATGAAT	Creation of the 1489 bp deletion allele
C-*crp*	F: GCGGAATTCATGGTTCTAGGTAAACCTCAA<br>R: CCCAAGCTTTTAGCGAGTACCGTAAACCAC	Amplification of 651 bp *crp* coding fragment
Crp	F: ACGTTATCCAATGGCGAATG<br>R: TACCGTGATCATGTGCACAG	Amplicons of 381 and 934 bp for identification of Δ*crp* and WT strains
Crp-RT-PCR	F: TAAACCTCAAACCGATCCAAC<br>R: TTCAAACAAGCCGAGTTCACC	Amplification of 223 bp for identification of *crp* gene intragenic region
pKD46	F: ATGACACCGGACATTATCCTG<br>R: TTTCAGCCAGTGCCTCGTC	Amplicons of 649 bp for identification of pKD46 plasmid recombinant strain
pCP20	F: TGCTCCACCCACAGGATGCT<br>R: ACACTGGCACCGCCGTTACT	Amplicons of 474 bp for identification of pCP20 plasmid recombinant strain
pBAD24	F: ATGCCATAGCATTTTTATCC<br>R: GATTTAATCTGTATCAGG	Amplicons of 798 bp for identification of complementing strain

Table S2. RT-qPCR Primers used in this study.

**Genes**	**Primer sequences (5′-3′)**	**Size (bp)**
16S	F: CGTGCTACAATGGCGTATACAGAGG<br>R: GCGATTACTAGCGATTCCGACTTCA	125
vmh	F: CCTGAGTTATTGGTTGGTGGTACGG<br>R: CGCCAGAGGTCGCTGTGATAGA	85
tlh	F: CCACGGCAGCAACTGAACCAT<br>R: GGACGATACCAACAGCGGACATAAG	109
epsM	F: TGCTTATCTGCAAGAGCGACAAGG<br>R: TGACTTCCACCACGCCATTTACTTT	83
epsL	F: ACCTGCTTGCTGCTTCCGATTG<br>R: CGCCACACTTGCTGTTCCACTAA	106
epsI	F: TCGTGAGGGTAGTGAGCAGATGG<br>R: TTTGAGCAGATTGTCGGCGGTTT	82
epsG	F: TGGCGGTTACATCAAGCGTCTG<br>R: CCATCAGCACCTAGAGTGAACACAT	108
epsF	F: CAAGTATTGGCGGCGGCAGAA<br>R: CACTCTGTTCACCACTGGCAATCA	118
epsE	F: TTCCTCTTCACTGCTTGGCGTATTG<br>R: TGCGTTGCTCTTTGTCTGCTTCA	95
epsC	F: GGACCTGACCGATCCGAATGTAATG<br>R: ATCATGTTGTTGACCATCACGCTCA	100
epsD	F: TCAATGCGGTGTCTAACGATTCCAA<br>R: TGCGGTTGAGCCAGTGATAACAG	125
vpsR	F: GTACATGGTCGCTCGTGACACTC<br>R: TGATGCGTTGCTGTTGGTATTGGA	88
vpsT	F: GCTCACACGAAAGTTAGCCCAAGA<br>R: CGATGCACCACTGCCGAGTAAC	133
rbmA	F: GGTTACATTCAACATGCGGCCAGTA<br>R: GCTTTCAGTCAAAGGTCCAGAAGGT	115
rbmA	F: GGCGTATTGAGATGACCTCCTGTG<br>R: TGTGCGTTCTGGTTATGCGATTGA	84
mshA	F: GACTCACTACCAACCGCAGAGC<br>R: GCAGATACATCAAGTACACCGCCAT	137
flaA	F: CAGCCGCTCGTGATCTGAAGTT<br>R: ACCGTTGATGTAAGTCGCCAATTCT	116
flrA	F: CTTCACGCCACGAGCAATCAATTC<br>R: TACAGGATGACCATACGCTCAACCA	96
flrC	F: GACTGCCTACGCCAACATTCAAGA<br>R: ACCACCGCATCACCATTGTCATC	150

fliA	F: CGGATGCTGAAGTGGCGAAGTT<br>R: ACGGTGAAATTGCGTCATCAGAAAC	129
fliD	F: TGAGTCCTGAAGAACGAGCCTACAT<br>R: GACGCCATCCAGCACAACCAT	143
flrB	F: GCGGATCAGTGATTTACAGCGTCTT<br>R: GCAGCAAGATTGGCGGCATACA	114
flgE	F: GCACAGCAGTTCCACGAAGGTT<br>R: CGCCGTTACGAGTCAGCTCATT	133
flhA	F: GGCACAGTTGATTGGTCACGAAGA<br>R: CAAGCGATAGTTGGTCAGGCACAA	104
flaB	F: CCGTGATACTGACTATGCTCGTGAA<br>R: AGATGGTGACTGCTTCGCTTGC	103
acfD	F: CCTGAATACGGCACCACTGAATAGC<br>R: ACCTCGGTAGCACCATCAACATTG	96
ompU	F: TCATCCGTGAGGCTGTCATTCAAG<br>R: TGGTCGCTTCAGTACCTGAGTTCA	150
hapA	F: GTGTGAACGCCGCTTGTGGAA<br>R: CGCCGCTACCGTGAATGTATAGAA	123

Table S3. Biochemical test results.

**Biochemical test**	**SCCF01**	**Δ*crp***
Ala-Phe-Pro-ARYLAMIDASE (APPA)	—	—
ADONITOL (ADO)	—	—
L-Pyrrolydonyl-ARYLAMIDASE (PyrA)	+	+
L-ARABITOL (IARL)	—	—
D-CELLOBIOSE (Dcel)	—	—
BETA-GALACTOSIDASE (BGAL)	+	+
H_2S PRODUCTION (H_2S)	—	—
BETA-N-ACETYL-GLUCOSAMINIDASE (BNAG)	+	+
Glutamyl Arylamidase pNA (AGLTp)	—	—
D-GLUCOSE (dGLU)	+	+
GAMMA-GLUTAMYL-TRANSFERASE (GGT)	—	—
FERMENTATION/ GLUCOSE (OFF)	—	—
BETA-GLUCOSIDASE (BGLU)	—	—
D-MALTOSE (dMAL)	+	—
D-MANNITOL (dMAN)	+	—
D-MANNOSE (dMNE)	—	—
BETA-XYLOSIDASE (BXYL)	—	—
BETA-Alanine arylamidase pNA (BAlap)	—	—
L-Proline ARYLAMIDASE (ProA)	—	—
LIPASE (LIP)	—	—

PALATINOSE (PLE)	−	−
Tyrosine ARYLAMIDASE (TyrA)	+	−
UREASE (URE)	+	+
D-SORBITOL (dSOR)	−	−
SACCHAROSE/SUCROSE (SAC)	−	−
D-TAGATOSE (dTAG)	−	−
D-TREHALOSE (dTRE)	+	−
CITRATE(SODIUM) (CIT)	−	−
MALONATE (MNT)	−	−
5-KETO-D-GLUCONATE (5KG)	−	−
L-LACTATE alkalinisation (ILATk)	+	−
ALPHA-GLUCOSIDASE (AGLU)	−	−
SUCCINATE alkalinisation (SUCT)	+	−
Beta-N-ACETYL-GALACTOSAMINIDASE (NAGA)	+	−
ALPHA-GALACTOSIDASE (AGAL)	−	−
PHOSPHATASE (PHOS)	+	−
Glycine ARYLAMIDASE (GlyA)	−	+
ORNITHINE DECARBOXYLASE (ODC)	+	−
LYSINE DECARBOXYLASE (LDC)	+	−
L-HISTIDINE assimilation (IHISa)	−	−
COURMARATE (CMT)	−	+
BETA-GLUCORONIDASE (BGUR)	−	−
O/129 RESISTANCE (O129R)	−	−
Glu-Gly-Arg-ARYLAMIDASE (GGAA)	+	−
L-MALATE assimilation (IMLTa)	−	−
ELLMAN (ELLM)	+	+
L-LACTATE assimilation (ILATa)	−	−
